# GAMEPLAN4CARE AND RESOURCES4CARE: PRELIMINARY OUTCOMES ASSOCIATED WITH TWO ONLINE DEMENTIA CAREGIVING PROGRAMS

**DOI:** 10.1093/geroni/igae098.2037

**Published:** 2024-12-31

**Authors:** Alan Stevens, Jinmyoung Cho, Jordan Reese, Gang Han, Marcia Ory

**Affiliations:** Baylor Scott & White Health, Belton, Texas, United States; Saint Louis University School of Medicine, Ballwin, Missouri, United States; Baylor Scott & White Health, Belton, Texas, United States; Texas A&M University, College Station, Texas, United States; Texas A&M University, College Station, Texas, United States

## Abstract

GamePlan4Care (GP4C) and Resources4Care (R4C) are online dementia caregiver programs. GP4C consists of rules-based conditional logic algorithms and real-time assessments of caregiving risks to tailor and prioritize educational and skills content. R4C provides a searchable database of vetted online educational and resources focused on dementia and family caregiving. The content of R4C was chosen to parallel GP4C educational content but does not include tailoring of content based on real-time caregiver assessments. Other program similarities were the self-paced format for caregivers’ engagement with brief educational videos and skill-building exercises and access to a Dementia Care Specialist to further support caregivers’ engagement. Two hundred forty family caregivers of persons living with dementia (PLWD) were randomly assigned to either GP4C or R4C. Nearly 70% of enrolled caregivers lived with a PLWD. Half were adult child/child-in-law caregivers (52%) and 39% were spousal caregivers. Preliminary results are reported on three standardized assessments conducted at baseline and a 6-month follow: Zarit Caregiver Burden Interview (ZBI), Positive Aspects of Caregiving (PAC), and Social Provision Scale (SPS). Caregivers assigned to either GP4C or R4C reported a significant (P<.05) reduction in ZBI and improvement in PAC after 6 months of self-paced engagement in GP4C and R4C. Caregivers in GP4C also reported a significant increase in perception of receiving guidance support from family or friends (SPS). Both GP4C and R4C show promise as online platforms for dementia caregivers. Feedback from participants of this study suggest the need for improved functionality and revision of content to better meet diverse caregiver needs.

